# IRS-Enabled Ultra-Low-Power Wireless Sensor Networks: Scheduling and Transmission Schemes

**DOI:** 10.3390/s22239229

**Published:** 2022-11-27

**Authors:** Hibatallah Alwazani, Anas Chaaban

**Affiliations:** School of Engineering, University of British Columbia, Kelowna, BC V1V1V7, Canada

**Keywords:** wireless sensor networks, intelligent reflecting surfaces, opportunistic beamforming, system outage probability, multi-user diversity, round robin, proportional fairness

## Abstract

Passive technologies, including intelligent reflecting surfaces (IRS), are gaining traction thanks to their ability to enhance communication systems while maintaining minimal cost and low complexity. They can assist a wireless sensor network (WSN) by achieving low power requirements for sensors and aid communication needs in many applications, for instance, environmental monitoring. In this paper, we propose an IRS-equipped WSN which describes sensors equipped with IRSs instead of active radio frequency (RF) electronics. The IRS sensor node (ISN) intercepts a dedicated signal from a power source such as a base station (BS) and modulates the transmission of that signal to an intended recipient. In order to enable multiple sensors to transmit to the receiver, we study opportunistic scheduling (OS) utilizing multi-sensor diversity while considering blind IRS operation, and compare it with round-robin (RR), proportional fairness (PF), and a theoretical upper bound. We study the effect of the choice of the number of IRS elements *N* and number of ISNs *L* on the average throughput of the system under OS. Finally, we provide pertinent comparisons for the different scheduling schemes and IRS configurations under relevant system performance metrics, highlighting different scenarios in which each scheme performs better.

## 1. Introduction

In light of recent advances in intelligent reflecting surfaces (IRSs), internet of things (IoT), and ultra-low power sensing devices, the convergence of these breakthrough technologies is bound to occur as innovative solutions to long-existing connectivity issues. It is universally acknowledged that the world is hurtling towards a data era unlike anything historically witnessed. The path towards Industry 4.0 is currently being paved, and it is materializing through wireless sensor networks (WSN) [1]. A WSN refers generally to a number of sensors occupying a sensing region to observe a certain physical phenomenon and carry on a specific task, such as surveillance, agriculture, monitoring, etc.

On the other hand, a paradigm shift in wireless communication is being introduced via smart radio environments, and enabled with intelligent reflecting surfaces [2]. Specifically, IRSs contain a large number of economical, passive, and scattering elements; each element can change the amplitude and/or shift the phase of impinging electromagnetic waves to achieve gains via reflected beamforming. An IRS reflects and scatters the incoming incident wave to achieve a desired objective. This objective may take the form of increasing the received power of the desired signal at a receiver, decreasing the power of interference, or increasing their ratio. This is achieved via beam-focusing (tuning the magnitude of the radiation pattern in a certain direction) or beam-steering (modifying the direction of the beam) [3]. Using beam-focusing or beam-steering, IRSs can aid wireless communications in a number of ways; for instance, IRSs can be used to enhance coverage range of wireless networks and provide significant rate improvements [4]. Furthermore, IRSs can enhance wireless powered communication networks in two phases: wireless information transfer and wireless energy transfer [5,6]. IRSs often compete with existing helper technologies such as relays; for instance, in [7] the authors used relays in a way similar to the power-saving approach described in this paper in order to prolong the lifetime of WSN via simultaneous wireless information and power transfer (SWIPT).

**Figure 1:**
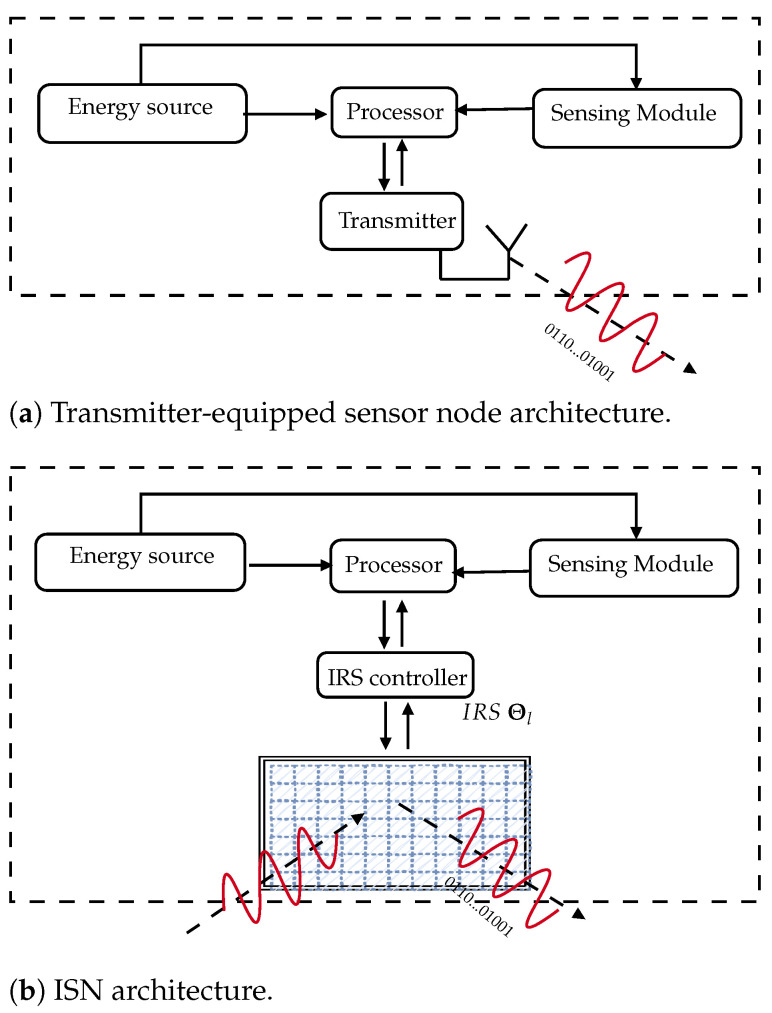
Two sensor node architectures. Common units are a sensing module, processor, and energy source. A transmitter unit is present in the transmitter-equipped sensor. Instead of a transmitter unit, we use an IRS controller and IRS in the ISN.

In certain WSN scenarios, sensors can be placed in remote, hazardous, or inaccessible terrain. In such cases, untangling the information source and the power source into two separate entities is desired to ensure that the sensors can monitor and survey the surroundings, thereby providing relevant real-time readings for a desired purpose while not equipped with a power supply dedicated to sending the readings. In this way, the realization of ISNs can reduce the need for battery replacement, minimize charging cost, and remove limitations due to inaccessible terrain. The ISN relinquishes the radio unit found in a transmitter-equipped sensor node, instead recycling and modulating intercepted waves from an outside dedicated power source. Figure 1a,b provides an illustrated comparison between an an abstract model of a traditional transmitter-equipped sensor node and an ISN. In Figure 1b, the IRS attached to the sensor can aid in transmitting information passively through backscattering. Backscattering has long existed and is ubiquitous in everyday life, as in the case of RFIDs [8].

Similar in operation to [5], the power resource is exported, in a sense, to a nearby base-station. However, unlike [5], in which the IRS was used as a helper to improve the energy harvesting and information transfer performance of an RF-equipped sensor node, the role of the IRS in this paper is to recycle the reference signal received from a base-station (BS) and modulate it in order to transfer the information from the sensor to which it is attached, i.e., perform backscattering communication. For clarity, an ISN includes a sensor node equipped with an IRS, although we often use the terms IRSs and ISNs interchangeably in this paper.

### 1.1. Challenges of WSNs

Although WSNs have emerged as a promising future technology to fulfill a purpose of an ultra-connected reality, a number of challenges have emerged alongside [9]. One such challenge is increasing the fault tolerance of sensor nodes for deployment in harsh environments. Another is the ability to scale the network, as the number of sensors is typically in the order of hundreds or thousands and these need to integrated into the network without overloading it. Perhaps the most constraining challenge is the reliance of WSNs on low-cost and resource-deprived equipment, which incurs several hardware limitations, including limited processor speed, small memory size, and low battery life. Limited power supply at the sensor due to low-cost consideration clashes with the surveillance-oriented nature of many sensor network applications. Indeed, the surveillance nature of sensor nodes automatically necessitates a long operational time; coupled with hazardous sensing scenarios negating battery replacement as a feasible response, there is a desperate need for an innovative solution.

### 1.2. How IRS Realizes Backscatter Communication

Backscatter communication is a technique that allows wireless nodes to communicate without an active radiofrequency (RF) component. It was first introduced in 1948, and is now gaining strides in internet of things (IoT) networks thanks to its low-power and spectrum-efficient advantages. It is comprised of two devices: a tag and a reader. The tag can harvest existing RF energy from received signals, then modulate and reflect those signals to convey information to the reader [10].

IRSs are often compared to relays and standard backscattering technology [11]. The former are differentiated because they have active transmission modules such as power amplifiers [2]. The latter is distinguished through its communications operation, wherein a reader transmits a signal to the a tag which then reflects a modulated signal back to the reader; this requires self-interference cancellation at the reader, and thus applications are mostly limited to short-range wireless sensor networks such as wearable IoT devices. On the other hand, there is a need for medium/long range wireless sensor networks which can monitor and observe large-scale phenomena at fine spatiotemporal resolutions, which can be achieved with the aid of IRSs. These sensor nodes are separated by long distances over a large geographical location, making the low power consumption of these sensors paramount for cost reduction. A fine spatial resolution of the sensing region requires a large number of sensors, each with its own set of observations. This incurs an issue in scheduling the ISN, which can be resolved using channelization, that is, a controlled access technique. For a WSN dedicated to environmental monitoring, systematic scheduling or channelization is a necessity, as multiple sensors transmit continuous streams of data. As such, random access protocols ( e.g., ALOHA, slotted ALOHA, and CSMA/CA), which work best with bursty traffic where users transmit on rare occasions, are bound produce to lower system performance [12]. Next, we discuss and compare transmission power and range in a typical WSN and an IRS-equipped WSN.

### 1.3. Problem Formulation

In this paper, we propose a novel IRS-equipped WSN system. Here, the novel IRS-equipped WSN can mitigate the issue of low battery lifetime in sensors. Instead of periodically replacing/recharging batteries embedded in the sensors, the IRS minimizes battery usage by relying on an outer power source for transmission while limiting the embedded energy source to local sensing and processing. A use case for this could be environment monitoring covering a large region with many nodes; in such a scenario, placing the sensor nodes is easy (i.e., by flying drones), while reaching the sensors to replace the batteries is an expensive and complex process. Another use case is in the event of limited transmission of economical off-the-shelf sensor nodes, in which case the transmission range could be increased without incurring any increase in the cost of the off-the-shelf sensors. In this work, we investigate the workings of this energy-efficient variant of a typical WSN.

First, we describe a scheme that caters to a system with blind operation of the IRSs in the sensor nodes. The rate of a BPSK input to a Gaussian channel is illustrated, and the system outage probability is defined for different scheduling schemes. The fairness/performance trade-off for different scheduling schemes in the system is explored via simulations. Finally, multi-sensor diversity in the system is exploited and the effect of increasing the number of ISNs is investigated. Our main contributions can be summarized as follows:

- We design a scheme for an IRS-equipped WSN with proper channel modelling and system parameterization.
- We analyze the performance of ssuch ISNs in terms of scheduling protocols, system outage probability, and rate analysis.
- We investigate dual IRS gains attained from IRS opportunistic beamforming and IRS information encoding using both continuous phases and practical discrete phases.
- Using simulations, we demonstrate how multi-sensor diversity can be exploited to overcome the blind operation of ISNs.

## 2. System Model

In this section, we describe the channel models used in the IRS-equipped WSN system. To clarify the mathematical symbols used, we list them in Table 1. The abbreviations used throughout the paper are listed in Table 2.

**Figure 2:**
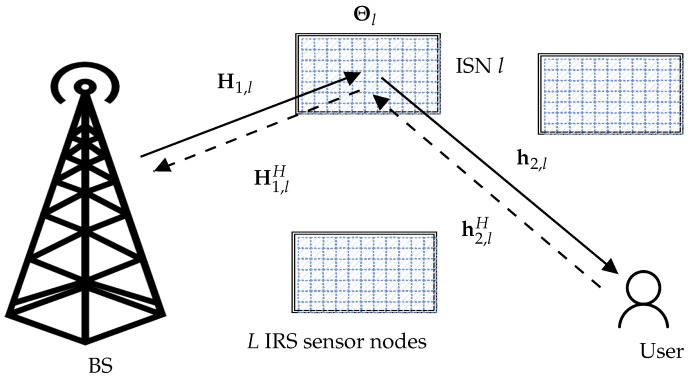
IRS-equipped sensor nodes (ISN)s in a WSN with *L* nodes. The solid lines represent downlink transmission, while uplinks are shown by the dashed lines.

Consider the system shown in Figure 2, with one single antenna user, a BS equipped with *M* antennas, and *L* IRS sensor nodes, hereinafter denoted as ISNs. In every ISN, a sensor unit or node is attached to an IRS that modulates the information from the sensor onto a carrier reference signal sent from the BS to the user. Each IRS is equipped with *N* IRS elements. The BS and all IRSs have no shared control links to assist in optimized transmission quality. Assuming a uniform linear array (ULA) at the BS and uniform planar array (UPA) at the IRSs, the known (due to the static nature of the BS and deployed sensors, and because we can place the ISNs in the LoS of the BS, the BS knows $H_{1,l}\forall l$) LoS BS-IRS *l* channel matrix $H_{1,l}$ can be written as

(1) $$\begin{array}{cc} \; & H_{1,l}=\sqrt{\beta_{1}^{l}}a_{l}b_{l}^{H}. \end{array}$$

Here, $\beta_{1}^{l}$ is the path loss factor for the BS-IRS *l* channel and $a_{l}\in C^{M}$ is the array response vector for the BS, while $b_{l}\in C^{N}$ is the array response vector for the IRS *l* and $H_{1,l}\in C^{M\times N}$. As we assume a ULA at the BS, the steering vector $a_{l}$ can be written as

(2) $$\begin{array}{c} a_{l}={\left[1,e^{-jkd_{BS}\;\cos\left(\phi_{l}\right)},\ldots ,e^{-jkd_{BS}\left(M-1\right)\;\cos\left(\phi_{l}\right)}\right]}^{T}, \end{array}$$

where $d_{BS}$ is the inter-antenna spacing, $k=2\pi /\lambda_{c}$ is the wave number, $\lambda_{c}$ is the carrier wavelength, and $\phi_{l}$ is the elevation angle-of-departure (AoD) from the BS to IRS *l* [13].

The IRS is envisioned to be a planar array of $N=N_{x}N_{z}$ elements, where $N_{x}$ denotes the number of horizontally placed elements and $N_{z}$ is the number of vertically placed elements. The array response vector $b_{l}$ for a UPA at IRS *l* is expressed as

(3) $$\begin{array}{c} b_{l}=b_{l,x}⊗b_{l,z} \end{array}$$

Here, ⊗ denotes the Kronecker product and

(4) $$\begin{array}{cc} \; & b_{l,x}={\left[1,e^{-jkd_{IRS}\;\sin\left(\varphi_{l}\right)\;\cos\left(\vartheta_{l}\right)},\ldots ,e^{-jkd_{IRS}\left(N_{x}-1\right)\;\sin\left(\varphi_{l}\right)\;\cos\left(\vartheta_{l}\right)}\right]}^{T}, \end{array}$$

(5) $$\begin{array}{cc} \; & b_{l,z}={\left[1,e^{-jkd_{IRS}\;\cos\left(\varphi_{l}\right)},\ldots ,e^{-jkd_{IRS}\left(N_{z}-1\right)\;\cos\left(\varphi_{l}\right)}\right]}^{T}, \end{array}$$

where $d_{IRS}$ is the inter-element spacing and $\vartheta_{l}\left(\varphi_{l}\right)$ denote the azimuth (elevation) angle-of-arrival AoA of the path from BS to IRS *l* [14]. We define the IRS *l* to the user channel as $h_{2,l}\in C^{N}$, where $h_{2,l}∼\mathrm{CN}\left(0,\beta_{2}^{l}I_{N}\right)$, $\beta_{2}^{l}$ is the path loss factor and $I_{N}$ is the N×N identity matrix. Assuming the direct link between the BS and user is obstructed due to high penetration losses, the end-to-end channel from the BS to the user through IRS *l* is provided by

(6) $$\begin{array}{cc} h_{l}\left(t\right) & =H_{1,l}\Theta_{l}\left(t\right)h_{2,l}=G_{l}v_{l}\left(t\right)∼\mathrm{CN}\left(0,\beta_{l}H_{1,l}H_{1,l}^{H}\right), \end{array}$$

where $\beta_{l}=\beta_{1}^{l}\beta_{2}^{l}$ is the total path loss factor for the cascaded IRS channel. Because $\Theta_{l}\left(t\right)$ is a diagonal matrix, we can rewrite $H_{1,l}\Theta_{l}\left(t\right)h_{2,l}$ as $G_{l}v_{l}\left(t\right)$, in which $G_{l}=H_{1,l}\mathrm{diag}\left(h_{2,l}\right)\in C^{M\times N}$. Each IRS element *n* has has a phase denoted by $\theta_{l,n}\left(t\right)$. The phase shift matrix $\Theta_{l}\left(t\right)\in C^{N\times N}$ is diagonal with elements containing the phase shifts of IRS *l*, and is defined as $\Theta_{l}\left(t\right)=\mathrm{diag}\left(v_{l}\left(t\right)\right)$ and $v_{l}\left(t\right)={\left[e^{j\theta_{l,1}\left(t\right)},\cdots ,e^{j\theta_{l,N}\left(t\right)}\right]}^{T}$, where the diag(·) operator transforms a vector into a diagonal matrix.

Note that we parameterize $\Theta_{l}$ with a time variable (t); this is because, as we shall see, $\Theta_{l}$ changes as a function of time in order to impart modulation onto the signal reflected from the IRS. The slow-varying channel $h_{2,l}$ that remains constant for time $\tau_{C}$ is called the coherence time, and corresponds to a number of symbols $T_{S}$ such that $\tau_{C}=T_{s}\tau_{S}$ and $\tau_{S}$ is the transmission time for one symbol. We call a coherence interval $\tau_{C}$ a frame which is indexed by a frame index $t_{f}$. Furthermore, the ISNs are blindly operated, i.e., they do not take channel variations into account when reconfiguring their respective phase shift matrices. Next, we describe and study a transmission scheme for the IRS-assisted WSN.

## 3. Transmission Scheme

Leveraging the objective of WSNs to have minimal network infrastructure and reduce operational costs, this work assumes that the ISNs are a completely additive technology without any control links between the ISNs and BS. Because the system is without backhaul links, there is no need to perform extensive channel estimation at the BS, as the IRS cannot utilize this information to reconfigure accordingly. Hence, we avoid the caveat of channel estimation in IRS-assisted systems, which scales prohibitively with the dimensions of the system (number of antennas at the BS *M*, number of IRS elements *N*, and number of ISNs *L*). Instead, we propose a centralized and controlled access technique by which the BS assigns and activates a particular ISN to begin transmission.

**Figure 3:**
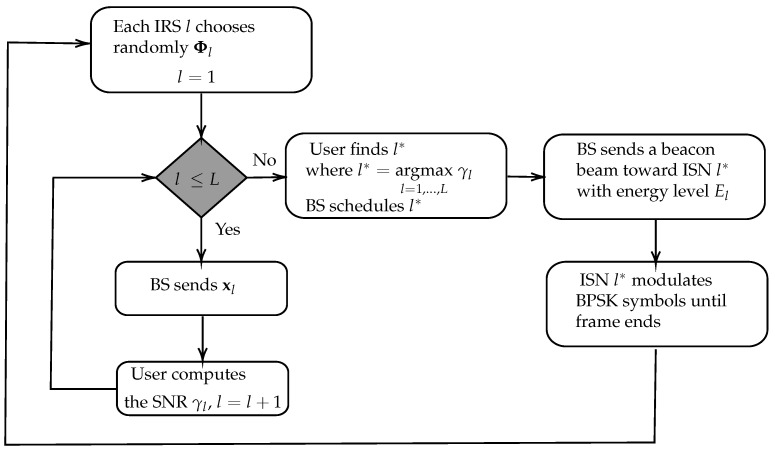
Flowchart for opportunistic scheduling in a certain frame index $t_{f}$.

**Figure 4:**
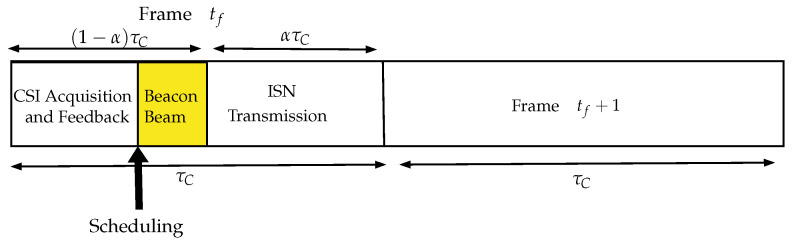
The sequence of scheduling and information transmission detailed for a particular frame $t_{f}$.

Scheduling sensors in a WSN requires a simple solution, especially considering the lack of control links between all the ISNs and the BS, as well as the lack of an RF front-end at the IRS. A scheduling algorithm is often implemented to manage the sensors in a collision-free manner [15]. The ISNs can be scheduled without any feedback using round-robin (RR) scheduling by having each take a turn sequentially. Other scheduling schemes require feedback, such as the OS scheme visualized in Figure 3. Below, we detail a general outline of this scheme, with this sequence illustrated in Figure 4:

1. In a frame $t_{f}$, the BS sweeps through all the ISNs with a known reference signal $x_{l}$, defined as
  (7) $$\begin{array}{c} x_{l}=\sqrt{P_{l}}f_{l}, \end{array}$$
  in which $f_{l}\in C^{M}$ is a beamforming vector designated for ISN *l* with unit norm $∥f_{l}{∥}^{2}=1$ and $P_{l}$ is the power allocated for the reference signal catering to ISN *l*. Moreover, each IRS pseudo-randomly chooses a phase configuration $\Phi_{l}$; thus, when the incident wave from the BS impinges on it, the IRS scatters it randomly towards the user.
2. The user computes the signal-to-noise ratio (SNR) $\gamma_{l}$ defined in (13) and feeds it back to the BS.
3. After a full sweep illuminating each ISN, the BS now has $\gamma_{l},l=1,\ldots ,L$, corresponding to each ISN. The BS then schedules the IRS according to a certain performance metric, such as throughput or fairness, using a beacon beam to awaken it for modulation.
4. The ISN begins modulating onto the reference wave using BPSK.

Note that the sweep through the ISNs is an overhead that scales with *L* (number of ISNs), and does not scale with *N* (number of IRS elements) or *M* (number of antennas at BS). In the following subsections, we look into the definition of asymptotic favorable propagation, the introduction of beacon beams, and the end-to-end transmission that enables this scheduling protocol.

### 3.1. Asymptotic Favorable Propagation

Because the BS has knowledge of $H_{1,l}\forall l$, the beamforming vector for a scheduled ISN *l* must direct the energy of the beam to concentrate on the location of IRS *l*. If the BS has a large enough number of antennas M≫L, it can transmit a beam in the null space of all other ISNs while beaming towards the scheduled ISN. Before defining the beam, we make use of the following lemma.

**Lemma 1.**  *A set of vector channels $a_{l}$, defined in (*2*) with distinct AoDs $\phi_{l}$s, satisfies asymptotic favorable propagation if $\left(kd\left(cos\left(\phi_{l}\right)-cos\left(\phi_{j}\right)\right)\;\left(\mathrm{mod}\;2\pi \right)\neq 0\right)$ for all l,j∈{1,⋯,L},l≠j, i.e.,*

(8) $$\begin{array}{c} \frac{1}{M}a_{l}^{H}a_{j}\to 0\;\mathrm{as}\;\;M\to \infty ,\;\forall l,j\in \left\{1,\cdots ,L\right\},l\neq j. \end{array}$$

**Proof.**  The proof is similar to [16], and is hence omitted. □

We exploit this property in our scheme as follows: the BS beamforms its transmitted signal such that the received signal power at a desired ISN is large, whereas the received signal power at all other ISNs is small. This is explained below through an example. Consider a case L=2 in which $H_{1,1}=\beta_{1}a_{1}b_{1}^{H}$ and $H_{1,2}=\beta_{2}a_{2}b_{2}^{H}$. If the BS sends $x_{1}\in C^{M}$, both ISNs receive

(9) $$\begin{array}{cc} y_{1} & =x_{1}^{H}H_{1,1}=\beta_{1}x_{1}^{H}a_{1}b_{1}^{H}, \end{array}$$

(10) $$\begin{array}{cc} y_{2} & =x_{1}^{H}H_{1,2}=\beta_{1}x_{1}^{H}a_{2}b_{2}^{H}. \end{array}$$

We aim to precode the transmitted reference signal $x_{1}$ defined in (7) for ISN 1 such that the effective channel to ISN 1 is maximized and the effective channel to ISN 2 is negligible. To this end, we choose the precoding vector as $f_{1}=\frac{a_{1}}{∥a_{1}∥}$ (maximum ratio transmission towards ISN 1), which maximizes the channel to ISN 1. In order to ensure that the channel to ISN 2 is minimal, we need $y_{2}=\beta_{1}x_{1}^{H}a_{2}b_{2}^{H}=0$. Thus, we need $\frac{a_{1}^{H}a_{2}}{∥a_{1}∥}=0$, or equivalently, $\frac{a_{1}^{H}a_{2}}{M}=0$, as $∥a_{1}∥=M$ (cf. (2)). Here, asymptotic favorable propagation (see Lemma 1) enters the picture. If the channels $a_{1}$ and $a_{2}$ satisfy asymptotic favorable propagation, then this condition is satisfied when *M* is large, and the effective channel from the BS to ISN 2 becomes negligible.

### 3.2. CSI Acquisition and Feedback

First, we denote the signal sent from the BS to ISN *l* as $x_{l}=\sqrt{P_{l}}\frac{a_{l}}{∥a_{l}∥}$. Thus, the user receives the following signal in time slot *t*:

(11) $$\begin{array}{c} Y\left(t\right)=x_{l}^{H}h_{l}\left(t\right)+x_{l}^{H}\sum_{j=1,j\neq l}^{L}h_{j}\left(t\right)+Z\left(t\right), \end{array}$$

where $h_{j}\left(t\right)=H_{1,j}\Theta_{j}\left(t\right)h_{2,j}$ as defined in (6), where $Z\left(t\right)∼\mathrm{CN}(0,\sigma^{2}$) is the noise at the user and $\sigma^{2}$ is the power of the noise.

The other IRSs receive negligible power from the BS due to favorable propagation, hence the second term in (11) is small; specifically, the signals from other ISNs are small, and are thus neglected. As such, (17) simplifies to

(12) $$\begin{array}{c} Y\left(t\right)=x_{l}^{H}h_{l}\left(t\right)+Z\left(t\right), \end{array}$$

The instantaneous effective SNR at the user through the cascaded *l*th IRS link is provided by (cf. (12))

(13) $$\begin{array}{c} \gamma_{l}=\frac{P_{l}{\left|f_{l}^{H}H_{1,l}\Phi_{l}h_{2,l}\right|}^{2}}{\sigma^{2}}={\left|f_{l}^{H}H_{1,l}\Phi_{l}h_{2,l}\right|}^{2}\gamma , \end{array}$$

where $\gamma =\frac{P_{l}}{\sigma^{2}}$. This initial CSI acquisition sweep generates $\gamma_{l}$s at the user. The user can use this information to schedule an ISN for transmission based on a scheduling scheme (detailed in Section 4), then send the index of the scheduled ISN to the BS. After that, the BS activates the ISN with a beacon beam to awaken it, which is explained next.

### 3.3. Activation and Beacon Beam

There is a need for system-level synchronization between the ISNs, the external power source (BS), and the recipient of information (user). This synchronization is crucial because it aids reliable information transmission without added frame errors. To realize this, we propose equipping the ISN with energy sensors that can detect the amount of energy a sensor receives from the BS. To activate the ISN, the BS sends a beacon beam towards it by transmitting a constant power signal for a duration of *T* symbols to awaken the ISN. The ISN is equipped with energy detectors that can measure the energy of the signal $E_{l}$. A beacon beam is thus defined as a wake-up beam aimed at a certain ISN with an energy level exceeding a certain threshold, i.e.,

(14) $$\begin{array}{c} E_{l}>E_{threshold}, \end{array}$$

To ensure a universal energy threshold $E_{threshold}$ for all ISNs, the power allocation in this scheme is a channel inversion in which

(15) $$\begin{array}{c} P_{l}=\frac{P}{\beta_{1}^{l}} \end{array}$$

to ensure equal reception of signal power at the ISNs.

When the beacon beam impinges on the scheduled ISN, the ISN is activated and begins to modulate the signal using the BPSK symbols by adding a common phase $\phi_{com}\in \left\{0,\pi \right\}$ across all *N* elements. Next, we detail how the IRS can send information to the user by piggybacking on the reference signal from the BS, then describe the received signal at the user.

### 3.4. ISN Transmission and Rate Analysis

When ISN *l* is activated by the beacon beam, it starts modulating the phase shift matrix $\Theta_{l}\left(t\right)$ as follows:

(16) $$\begin{array}{c} \Theta_{l}\left(t\right)=\Phi_{l}X_{l}\left(t\right), \end{array}$$

where $X_{l}\left(t\right)\in \left\{-1,1\right\}$ according to a BPSK signal and $\Phi_{l}=\mathrm{diag}\left(e^{j\theta_{l,1}},\cdots ,e^{j\theta_{l,N}}\right)$. To highlight the information modulation, (12) becomes

(17) $$\begin{array}{c} Y=\sqrt{P_{l}}\left(f_{l}^{H}H_{1,l}\Phi_{l}h_{2,l}\right)X_{l}\left(t\right)+Z, \end{array}$$

Note that a phase shift of π does not deter the effective channel magnitude, as $∥G_{l}v_{l}{∥}^{2}={∥G_{l}v_{l}e^{j\pi }∥}^{2}$. Thus, this modulation scheme does not change the signal-to-noise ratio; moreover, $\Phi_{l}\forall l$ is constant during the frame, which in turn means that the scheduling decision remains valid.

For a BPSK input for (17), we know that $X_{l}\left(t\right)\in \left\{-1,1\right\}$ with equal probability. The theoretical achievable rate formulated by Shannon (in bits per transmission) is then [17]

(18) $$\begin{array}{c} R_{l}^{th}=I\left(X_{l};Y\right). \end{array}$$

Using a binary capacity-approaching code, such as LDPC codes or polar codes, the achievable rate of such a scheme can be written as

(19) $$\begin{array}{c} R_{l}=1-H\left(p_{e}\right), \end{array}$$

where $H(p_{e})$ is the binary entropy function and $p_{e}$ is the cross-over probability of the binary symmetric channel. Note that for BPSK we have

(20) $$\begin{array}{c} p_{e}=Q\left(\sqrt{2\gamma_{l}}\right) \end{array}$$

Here, the Q function is

(21) $$\begin{array}{c} Q\left(z\right)=\int_{z}^{\infty }\frac{1}{\sqrt{2\pi }}e^{\frac{-x^{2}}{2}}dx. \end{array}$$

The achievable rate expression in (19) is used in subsequent calculations for outage rate analysis. Next, we define both the direct and feedback-based scheduling schemes utilized in this work for comparison with OS.

## 4. Scheduling Schemes

In Section 3, we described the transmission scheme when assuming ISN *l* has been scheduled to transmit. In this section, we describe different scheduling schemes that can be used within this system.

### 4.1. Opportunistic Scheduling

In OS, we schedule in a frame index $t_{f}$ an ISN $l^{*}$ which has the best overall channel; formally,

(22) $$\begin{array}{c} l^{*}=\underset{l=1\ldots L}{\mathrm{argmax}}\;\left|f_{l}^{H}H_{1,l}\Phi_{l}h_{2,l}\right|, \end{array}$$

where $\gamma_{l}$ is as defined in (13). This scheduling depends on SNRs from all the ISN links choosing the link that performs best, which occurs on the user’s end when the index of the scheduled ISN is sent to the BS. If the number of ISNs *L* grows large, an IRS becomes more likely to be scheduled with a pseudo-random configuration close to its optimal configuration for a given channel realization; thus, the system rides the peaks of the fading channels [18]. This phenomenon arises from the notion of multi-user diversity; in a system with many random links that vary independently, there is likely to be a link which provides the user with overall performance near the peak at any one time [19].

In this greedy method of scheduling, it may happen that the BS chooses the same ISN in every time slot if it continuously has the highest SNR compared to other links, which compromises fairness. The next scheduling scheme addresses this fairness issue.

### 4.2. Round-Robin

To achieve complete fairness, RR scheduling permits ISNs to take turns transmitting. For a particular frame index $t_{f}$, the scheduled ISN $l^{*}$ can be written as

(23) $$\begin{array}{c} l^{*}=1+t_{f}\;\left(\mathrm{mod}\;L\right). \end{array}$$

Because RR scheduling is channel-independent, CSI feedback is not needed in this case and the transmission time is not reduced by the time spent on CSI feedback. We define α∈{0,1} as the fraction of time reserved for information transmission, while 1−α is the fraction reserved for CSI feedback. In RR, α=1, as no CSI is needed at the BS in order to schedule. However, this scheduling scheme is suboptimal, as it does not take advantage of all the degrees of freedom in the system.

Furthermore, we define a variation of this scheme called RR with optimal phases, which we use as a baseline for simulations; in this scheme, we assume that backhaul links exist and that IRSs choose optimal phases while scheduled in an RR fashion. This scheme is helpful to examine the advantages of scheduling vs. passive beamforming at the IRSs.

The next scheduling scheme lies between RR and OS, and can be tweaked in order to achieve appropriate fairness/performance criteria.

### 4.3. Proportional Fairness

Proportional fairness (PF) is a scheduling scheme that finds a middle ground between two competing interests, namely, optimizing network throughput and maintaining a minimal service for all network nodes. To find the scheduled ISN $l^{*}$ for a frame index $t_{f}$, we define the average throughput of an ISN ${\bar{T}}_{l}\left(t_{f}\right)$ over a window of time $t_{w}=F\tau_{C}$, where *F* is the number of frames, as follows: [19]

(24) $$\begin{array}{c} {\bar{T}}_{l}\left(t_{f}+1\right)=\left\{\begin{array}{c} \left(1-\frac{1}{t_{w}}\right){\bar{T}}_{l}\left(t_{f}\right)+\frac{1}{t_{w}}R_{l}\left(t_{f}\right),\;l=l^{*}\\ \left(1-\frac{1}{t_{w}}\right){\bar{T}}_{l}\left(t_{f}\right),\;l\neq l^{*},l=1,\ldots ,L \end{array}\right. \end{array}$$

Thus, the BS calculates a long-term average over a window of time $t_{w}$. Scheduling occurs in every frame $t_{f}$ as through the ISN with highest ratio of rate to average throughput, i.e.,

(25) $$\begin{array}{c} l^{*}=\underset{l=1\ldots L}{\mathrm{argmax}}\;\frac{R_{l}\left(t_{f}\right)}{{\bar{T}}_{l}\left(t_{f}\right)} \end{array}$$

### 4.4. Coherent Beamforming Benchmark

Previous scheduling schemes presume no control links; hence, the IRS can only change configuration pseudo-randomly every frame $t_{f}$, as opposed to coherently beamforming in order to increase the received signal strength at the user. In the presence of control links between the BS and the IRSs, the IRS can be configured to the optimal configuration towards the scheduled user. This leads to the coherent beamforming benchmark that we present here. In this benchmark, we assume the IRS in the ISN can find its optimal configuration vector denoted by $v_{l}^{*}=\mathrm{vec}\left(\Phi_{l}^{*}\right)$ at ISN *l* by choosing to enhance the collective channel gain, i.e., maximizing $∥h_{l}{∥}^{2}$ as provided in (6).

The optimization problem is defined as

(26) $$\begin{array}{cc} \left(P0\right)\;\max_{v_{l}} & \;∥G_{l}v_{l}{∥}^{2} \end{array}$$

(27) $$\begin{array}{cc} \; & s.t.\;|v_{l,n}|=1\forall n,\forall l, \end{array}$$

**Theorem 1**  ([3]). *The optimal solution of (P0) for the beamforming vector of IRS l is provided as*

(28) $$\begin{array}{c} v_{l}^{*}=e^{j∠\mathrm{diag}\left(h_{2,l}^{H}\right)b_{l}}, \end{array}$$

which can be written for each element as $v_{l,n}^{*}=e^{j(-∠h_{2,l,n}+∠b_{l,n})}$, where $h_{2,l,n}$ and $b_{l,n}$ are the n*th* elements of $h_{2,l}$ and $b_{l}$, respectively.

Here, we find the optimal configuration $v_{l}^{*}$ for each IRS, after which the BS schedules the ISN with the maximum SNR in the system:

(29) $$\begin{array}{c} l^{*}=\underset{l=1\ldots L}{\mathrm{argmax}}\;\left|f_{l}^{H}H_{1,l}\Phi_{l}^{*}h_{2,l}\right|. \end{array}$$

In a way, this is similar to OS, except with $\Phi_{l}^{*}$ instead of a pseudo-random configuration. Next, we define an outage in an IRS-equipped WSN.

In the benchmark case, the BS has perfect CSI and there are backhaul links between the BS and all the ISNs. Thus, we can assign $\Theta_{l}^{*}=\Phi_{l}^{*}X_{l}\left(t\right)$ for a scheduled ISN *l*. Sending one bit at a time from ISN *l* and assuming a continuous phase $\theta_{n}$, we assign the same information-carrying phase shift for each element in the IRS while negating the overall cascaded channel phase uniquely for each IRS, such that

(30) $$\begin{array}{c} \theta_{n}^{*}=-∠h_{2,l,n}+∠b_{l,n}+\left\{0,\pi \right\}; \end{array}$$

with a BPSK modulation scheme at the IRS, the common phase $\phi_{com}$ is set to 0 for all *N* elements in the case of a 0 information bit and to π in case of 1 information bit. With an optimal configuration at the IRS and utilizing (17), the user obtains

(31) $$\begin{array}{cc} Y_{l} & =\sqrt{P_{l}}\left(f_{l}^{H}H_{1,l}\Phi_{l}^{*}\left(t\right)h_{2,l}\right)X_{l}\left(t\right)+Z_{l}, \end{array}$$

(32) $$\begin{array}{cc} \; & =\sqrt{P_{l}}\left(f_{l}^{H}a_{l}\right)b_{l}^{H}\Phi_{l}^{*}\left(t\right)h_{2,l}X_{l}\left(t\right)+Z_{l} \end{array}$$

(33) $$\begin{array}{cc} \; & =\sqrt{P_{l}}\left(f_{l}^{H}a_{l}\right)\sum_{n}b_{l,n}h_{2,l,n}e^{j\theta_{n}^{*}}X_{l}\left(t\right)+Z_{l} \end{array}$$

(34) $$\begin{array}{cc} \; & =\sqrt{P_{l}}∥a_{l}∥\sum_{n}|b_{l,n}\left|\right|h_{2,l,n}|X_{l}\left(t\right)+Z_{l} \end{array}$$

where $f_{l}=\frac{a_{l}}{∥a_{l}∥}$.

## 5. Outage

We assume a fixed-rate transmission scheme where all ISNs transmit at a target rate $R_{target}$. Note that this is the coding rate; the actual rate in bits per second *s* needs to be scaled by the probability of a given sensor being scheduled. A sensor is scheduled in each frame based on the selected scheduling algorithm. If the channel of the scheduled sensor can support the rate $R_{target}$, transmission is successful. Otherwise, transmission errors may occur, and the system is said to be in outage.

The outage probability $P_{out,l}$ of a sensor *l* during a time frame in which it is scheduled for transmission is written as

(35) $$\begin{array}{c} P_{out,l}=Pr\left(R_{l}\leq R_{target}\right), \end{array}$$

where $R_{l}$ is the rate that can be supported by the channel from ISN *l* to the user, which changes according to the channel. Next, we present the system outage probability for the different scheduling schemes.

The RR scheduling system outage probability can be constructed from ${\bar{P}}_{out,l}$ as follows [20]:

(36) $$\begin{array}{c} {\bar{P}}_{out,rr}=\frac{1}{L}\sum_{l=1}^{L}P_{out,l} \end{array}$$

For OS, we have a smaller system outage probability due to selecting the best ISN to transmit at a given time. The system outage probability of OS is defined as [20]

(37) $$\begin{array}{cc} {\bar{P}}_{out,os} & =Pr(\underset{l=1,\ldots ,L}{\mathrm{argmax}\;}R_{l}\leq R_{target}) \end{array}$$

(38) $$\begin{array}{cc} & =Pr(R_{l}\leq R_{target},\forall l) \end{array}$$

(39) $$\begin{array}{cc} & =\prod_{l=1}^{L}Pr\left(R_{l}\leq R_{target}\right) \end{array}$$

(40) $$\begin{array}{c} =\prod_{l=1}^{L}P_{out,l} \end{array}$$

Finally, for the system outage probability of PF scheduling, we have

(41) $$\begin{array}{c} {\bar{P}}_{out,pf}=Pr\left(R_{l^{*}}\leq R_{target}\right), \end{array}$$

where $l^{*}$ is chosen as in (25).

Here, we look into two extreme cases: as the time window $t_{w}$ approaches infinity, we obtain ultimate fairness, with each sensor being scheduled uniformly on average; thus, we have the same expression as RR (36). On the other hand, for $t_{w}=1$, we take into account only the current SNR values in the system and reverting back to OS (40). Finally, for the range of $t_{w}$ values, we obtain a weighted sum of the two Equations (36) and (40), which can be considered as a trade-off between OS and RR. Defined below is an approximation of the PF system outage probability, in which λ∈[0,1] and λ can be some monotonic inverse function of $t_{w}$, provided as follows:

(42) $$\begin{array}{c} {\bar{P}}_{out,pf}\approx \lambda {\bar{P}}_{out,os}+\left(1-\lambda \right){\bar{P}}_{out,rr}=\lambda \prod_{l=1}^{L}P_{out,l}+\left(1-\lambda \right)\frac{1}{L}\sum_{l=1}^{L}P_{out,l}. \end{array}$$

## 6. Results

The simulation parameters are provided in Table 3. We consider a BS located at (0,0) m, *L* ISNs deployed on an arc of radius *r* randomly chosen between 10 m<r<20 m and a sector angle $\theta_{sec}$ between $-\pi /6<\theta_{sec}<\pi /6$ with respect to the BS, and a single antenna user located at (25,0) m. Thus, the ISNs are distributed within a difference of sector areas such that $A=\frac{(20^{2}-10^{2})\pi }{6}\;m^{2}$ and the simulated network area $A_{network}$, including the BS, user, and ISNs, is $A_{network}=\frac{20\pi }{3}\times 25\;m^{2}\approx 524\;m^{2}$. Path loss factors are computed at 2.5 GHz for the 3GPP Urban Micro (UMi) scenario from TR36.814 (cf. [21] Section V).

Generally, the coverage area of the IRS-equipped WSN depends on the constrained transmitting power of the BS, wavelength of the carrier $\lambda_{c}$, and number of IRS-equipped sensors. In this layout, the number of elements of the IRS is *N* and the size and spacing of the elements depends on $\lambda_{c}$. Hence, the size of the IRS is in the order $\lambda_{c}^{2}N$. If we assume a carrier frequency of 30 GHz which is equivalent to $\lambda_{c}=1$ cm and N=32, then the area of each IRS is approximately $A_{IRS}=32$ cm^2^.

**Figure 5:**
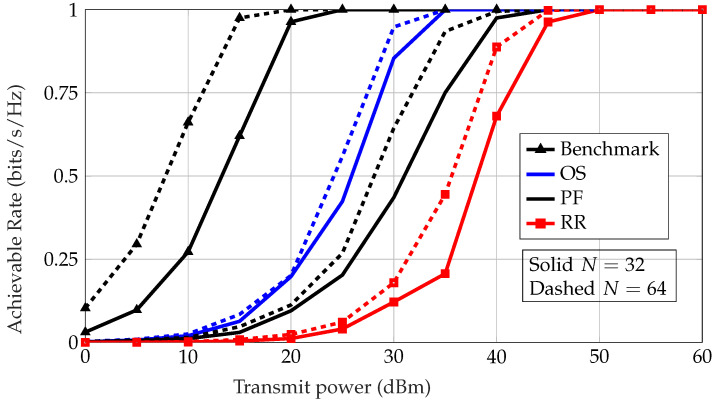
Rate vs. transmitting power *P* for different scheduling protocols.

In Figure 5, we plot the rate of the scheduled sensor under different scheduling protocols. First, the benchmark is defined as scheduling the ISN with the largest SNR under an an optimal phase configuration at the IRS while having perfect CSI. This is not achievable in practice in this system unless there are control links between the IRS and BS. Assuming that the time for feedback from the user to the BS is negligible, it is apparent that OS performs best, followed by PF, then RR. Note that for a larger number of elements *N* at the IRS there is an increase in performance thanks to the resulting increase in channel gain.

**Figure 6:**
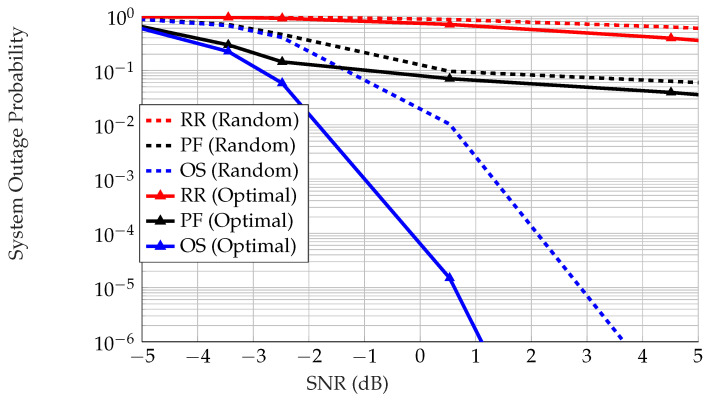
System outage probability vs. average SNR for all ISNs. The system outage probabilities for the OS, PF, and RR scheduling schemes are calculated according to Equations (36), (40), and (42), respectively.

The significance of using an optimal configuration of IRS $\Theta_{l}^{*}$ in each scheduled ISN versus a random configuration $\Theta_{l}^{rand}$ is showcased in Figure 6, where it can be seen that if there is a control link between the BS and ISNs and an optimal phase configuration is deployed for the cascaded channel, we obtain better performance. Note that OS performs better in terms of outage than RR and PF with both the random phase and optimal phase configurations. The reason for this is that OS targets enhanced performance, which is equivalent to minimizing the system outage probability. Because OS searches for the ISN least likely to be in outage and schedules it, system outages are less likely overall than in other schemes, such as RR and PF, which target fairness between ISNs instead of overall performance. This highlights the significance of the OS protocol.

Next, we focus on the fairness metric in the proposed scheduling schemes. For instance, OS chooses the ISN with the best channel quality during each frame. This approach can be greedy, as weak ISNs may never be scheduled. On the one hand, the system throughput is maximized, while on the other fairness may be compromised. To ensure fairness amongst ISNs, a PF scheme can be implemented. PF caters particularly to ISNs with relatively weaker channel quality, as it takes into consideration the throughput of each ISN within a certain window of time $t_{w}$. By tuning $t_{w}$, we can enhance fairness, albeit by compromising system throughput.

**Figure 7:**
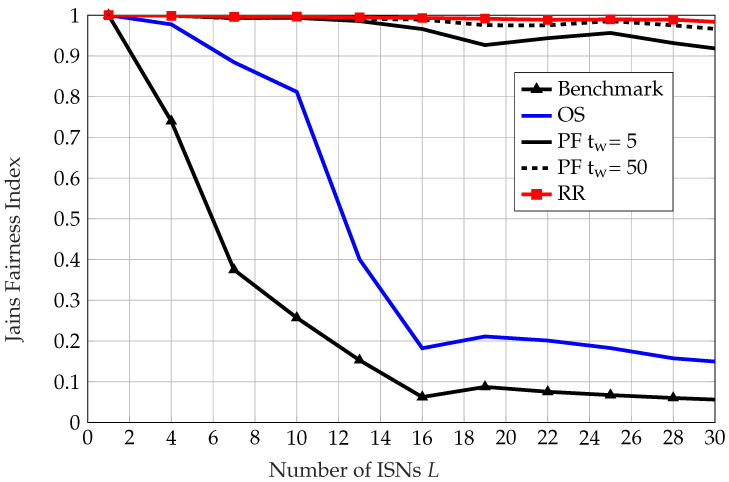
Jain’s fairness index as defined in (43) vs. number of ISNs *L*.

To highlight the fairness aspect, in Figure 7 we plot Jain’s fairness index, represented as

(43) $$\begin{array}{c} J\left(t\right)=\frac{{\left[\sum_{l=1}^{L}T_{l}\left(t\right)\right]}^{2}}{L\sum_{l=1}^{L}T_{l}{\left(t\right)}^{2}}, \end{array}$$

versus the number of ISNs *L* in the system, where $T_{l}$ is the throughput at frame $t_{f}$ and $l^{*}$ denotes the index of a scheduled ISN, defined as

(44) $$\begin{array}{c} T_{l}\left(t_{f}\right)=\left\{\begin{array}{c} T_{l}\left(t_{f}-1\right)+R_{l}\left(t_{f}\right),\;l=l^{*}\\ T_{l}\left(t_{f}-1\right),\;l\neq l^{*},l=1,\ldots ,L \end{array}\right. \end{array}$$

Note that $\frac{1}{L}\leq J\left(t\right)\leq 1$, in which 1 indicates best case for fairness when $T_{l}\left(t\right)=T$.

It be seen in Figure 7 that RR and PF have the best fairness performance among all other multi-user scheduling algorithms, as the others focus on ISNs with better channel quality and forgo fairness between the sensors. Moreover, it can be seen that the fairness indexes of PF and RR do not decrease with increasing number of ISNs, while those of OS and the benchmark decrease, as more ISNs indicate more competition and unfairness in the system. Furthermore, with a larger time window of $t_{w}=50$, PF approaches the fairness of RR scheduling and achieves higher fairness than with a smaller time window of $t_{w}=5$. Finally, it can be seen that the benchmark with OS results in worse fairness performance than OS under a random phase shift matrix.

**Figure 8:**
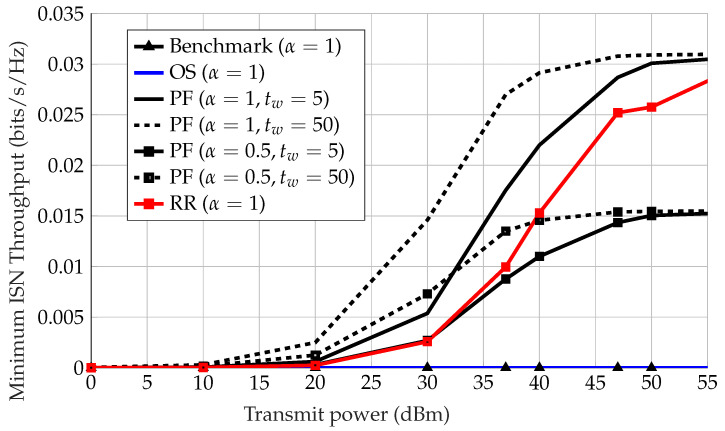
Minimum ISN throughput versus transmitting power for different scheduling protocols.

The minimum throughput $\tilde{T_{l}}\left(t_{f}\right)$ of the system, defined as

(45) $$\begin{array}{c} \tilde{T_{l}}\left(t_{f}\right)=\min_{l=1\ldots L}\;T_{l}\left(t_{f}\right), \end{array}$$

is plotted in Figure 8 to show that PF can be used to ensure fairness; furthermore this fairness can be adjusted by tuning the time window $t_{w}$ of past transmissions. The benchmark and OS rarely schedule the worst ISN in the system; thus, their minimum throughput is zero.

Moreover the uniform RR scheduling technique performs better than PF for the minimum throughput under certain transmitting powers and α, which is the fraction of time reserved for information transmission. SNR feedback for all the ISN links is only necessary for both PF and OS, as these rely on the varying random channels; RR does need such feedback from the user to the BS, meaning that more time can be allocated for information transmission. This is captured through α. Here, α∈[0,1] is a fraction of the time reserved for actual transmission and scales inversely according to *L*. For both PF and OS, the fraction of α is less than 1, as there is indeed time (negligible though it may be according to IEEE 802.11 standards) for SNR feedback from the user for all sensor links involved in the network. On the other hand, for RR, which is a channel-independent scheduling scheme, α=1.

**Figure 9:**
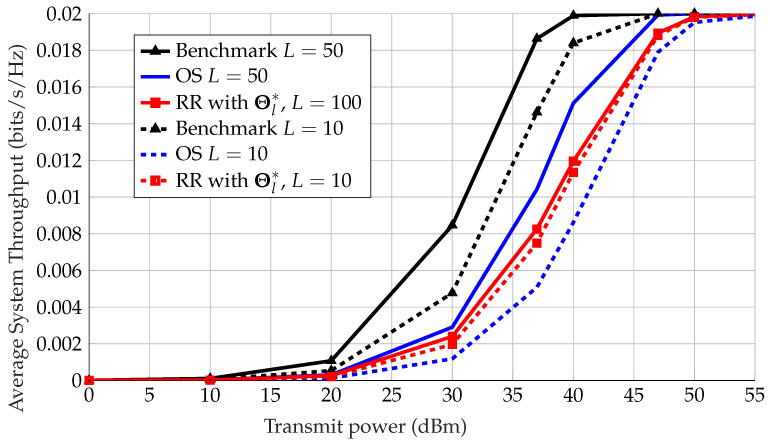
Average system throughput vs. transmitting power for optimal IRS configuration $\Theta_{l}^{*}$ with greedy scheduling, OS with random phases, and optimal IRS configuration $\Theta_{l}^{*}$ with RR scheduling, where N=8.

In Figure 9, which shows the average throughput of the system versus the transmit power, we assume that *M* is sufficiently large to ensure asymptotic favorable propagation (recall Lemma 1). We can observe the impact of the number of ISNs *L* on system performance under OS, with multi-user diversity being improved with a larger number of sensors. Moreover, note that for RR the impact of *L* is negligible with optimal choice of phases. This is because each sensor takes a turn to transmit, and having more sensors take turns, even if are optimal, plays virtually no role on the average throughput for the system due to the randomness of the channels. However, this is not the case for the benchmark; it can be seen that when *L* is larger the average throughput is improved, which is because we are maximizing over a larger sample from the same channel distribution, and have a higher chance of finding a stronger channel.

For L=100, OS with random phases at the IRSs surpasses RR scheduling with optimal phases, which is due to opportunistic beamforming and multi-sensor diversity gains being exploited [18]. Under this setup, we can conclude that the choice of scheduling is more significant than the choice of phases at the IRSs. On the other hand, for L=10, OS gains are smaller, as there is less multi-sensor diversity; hence, OS performs worse than RR with optimal phases.

**Figure 10:**
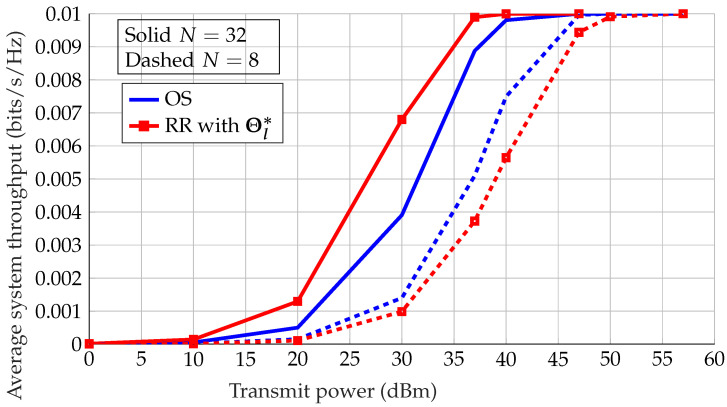
Average system throughput vs. transmitting power for OS with random phases and RR scheduling with optimal IRS configuration $\Theta_{l}^{*}$, where L=100.

Figure 10 shows the effect of the number of IRS elements *N* in the system on the performance of OS. A larger number of IRS elements *N* places a higher importance on the choice of the optimal phases. The search space of phases grows exponentially with *N*, and the probability of randomly choosing a configuration close to its optimal configuration decays exponentially; thus, multi-sensor diversity does not occur, and as a result RR with optimal phases outperforms OS in this case. We can conclude that OS gains are highlighted for smaller *N*, and that OS is virtually ineffective for larger *N*.

**Figure 11:**
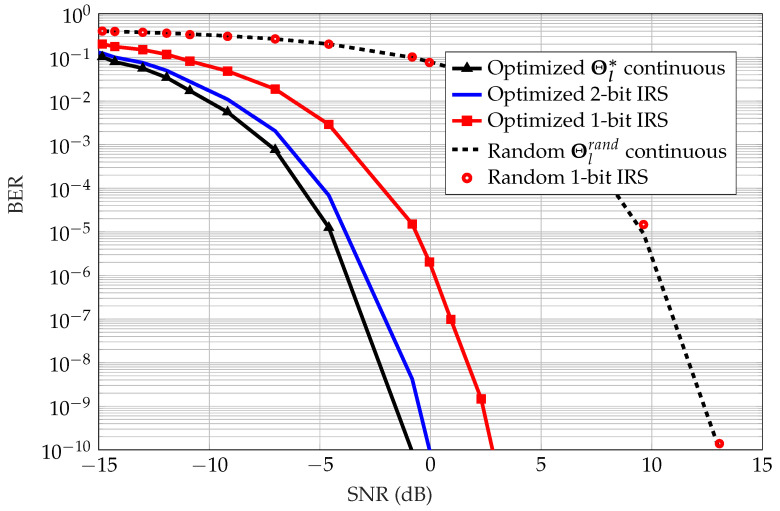
Bit error rate versus SNR for a single scheduled ISN transmission with different IRS phase resolutions and different phase configurations.

In practice, the phase shifts at the IRSs are from a discrete set. In this work, we assume that we are working with continuous phases, yet show the advantage of our work in the case of practical IRSs with discrete phases. For instance, Figure 11 shows the close performance between the BER of the continuous-phase IRS compared to a one-bit IRS that can only choose phases from a set $S_{1}=\left\{0,\pi \right\}$ or a two-bit IRS that can only choose phases from a set $S_{2}=\left\{0,\pi /2,\pi ,\frac{3\pi }{2}\right\}$. The way to choose quantized phases is to consider the desired phase shift vector $v_{l}^{*}$ for ISN *l* and choose from the set of feasible phases; for instance, for a one-bit IRS, each element can be chosen such that

(46) $$\begin{array}{c} \theta_{n}^{q,*}=\underset{\theta_{n}^{q,*}\in S_{1}}{\arg\;\min}{∥\theta_{n}^{*}-\theta_{n}^{q,*}∥}_{2} \end{array}$$

In addition, it can be seen that quantization effects are noticeable when the IRS is configured optimally. On the other hand, quantization minutely affects the BER with random phases chosen at the IRS. Because random phases are deployed when using OS, it is apparent that assuming practical quantization in the system has no adverse effect on the resulting BER, which is an advantage. In the next section, we compare a transmitter-equipped sensor and an ISN in terms of power consumption and range.

## 7. WSN Transmission Power and Range

The lifetime of a WSN compared to the lifetime of an IRS-equipped WSN boils down to a comparison between a scheduled sensor node with a transmitter unit and a scheduled ISN; the architecture of each is shown in Figure 1a,b. Because these units may be common, it is redundant to compare the energy consumption models of such units. Instead, we compare the energy consumption of the transmitter with the energy consumption of the IRS controller and IRS. Thus, it suffices to compare the power per symbol $P_{s,tx}$ for the transmitter under the transmitter-equipped sensor unit with the power per symbol in the ISN. If the power per symbol per IRS element is $P_{s,ISN}$, then the power per symbol for the whole ISN is $NP_{s,ISN}$, as all *N* elements are changing phase-shift according to the IRS controller in order to modulate the symbol. The transmitter-equipped sensor can be equipped with long-range wide area network (LoRaWAN) technology, which is popular in WSNs and can reach long transmission distances (around 10 km) with $P_{s,tx}=100$ mW [22]. On the other hand, existing works [23,24] have reported a power of $P_{s,ISN}=5$ mW for controlling an IRS element. For N=10, this amounts to a total of 50 mW, which may well decrease with future development of IRS technologies. Moreover, the range for this IRS-equipped WSN is not restricted by the ISN equipment. Instead, it is determined by the transmitting power of the BS, which is in the order of watts and is not battery-limited. This is compared to the transmitter-equipped sensors nodes, which are battery-limited and have transmitting power in the order of milliwatts. These two aspects combine to make ISNs suitable for scenarios with power and range challenges.

As a proof of concept, we focus on comparing the lifetime and range of an ISN with a LoRa sensor node, with the specifications provided in in Table 4, following the use case in [22]. For simplicity, we highlight the main sensor tasks of sensing, processing, and transmission, as illustrated in Figure 1a.

**Figure 12:**
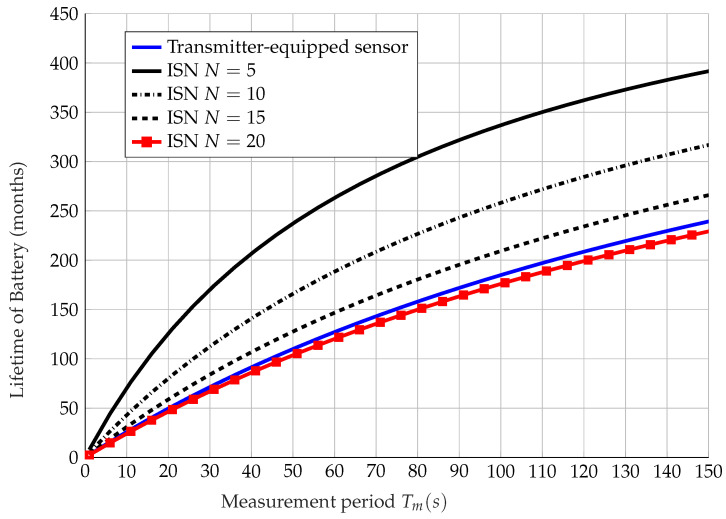
Battery lifetime of a single sensor node with an IRS (with different element number *N*) and without an IRS vs. measurement period $T_{m}$.

Figure 12 considers and compares the lifetime of a transmitter-equipped LoRa sensor battery with an ISN with N=5,10,15,20 elements against the measurement period with the battery characteristics described in Table 5. A measurement period describes a period in which the sensor is sensing, processing, and transmitting, and is sleeping for the remaining amount of time. In a measurement period $T_{m}$, the battery is sleeping for a time $T_{s}$ and active the rest of the time $T_{a}$, in which $T_{a}$ is found as

(47) $$\begin{array}{c} T_{a}=T_{tr}+T_{sen}+T_{proc}, \end{array}$$

where $T_{tr},T_{sen}$, and $T_{proc}$ are defined in Table 4 and $T_{s}=T_{m}-T_{a}$. Furthermore, the lifetime of the battery *L* for the sensor node can be calculated as [25]

(48) $$\begin{array}{c} L=E_{battery}/I_{avg} \end{array}$$

in hours, where $I_{avg}$ is the average current consumption of the sensor node. The energy in the battery is calculated as $E_{battery}=V_{b}C=3.3\times 950$ mJ and the $I_{avg}$ for the transmitter-equipped sensor is

(49) $$\begin{array}{cc} I_{avg} & =\frac{1}{T_{m}}\left(I_{tr}T_{tr}+I_{sen}T_{sen}+I_{proc}T_{proc}+I_{s}T_{s}\right) \end{array}$$

where $I_{tr}=\frac{92.4}{3.3}$ mA, $I_{sen}=\frac{10.5}{2}$ mA, $I_{proc}=\frac{1.8}{3.3}$ mA, and $I_{s}$ is found in Table 5. For the ISN, the average current consumption is calculated as

(50) $$\begin{array}{cc} I_{avg,ISN} & =\frac{1}{T_{m}}\left(NI_{ISN}T_{tr}+I_{sen}T_{sen}+I_{proc}T_{proc}+I_{s}T_{s}\right), \end{array}$$

where $I_{ISN}=\frac{P_{s,ISN}}{V_{b}}=\frac{5}{3.3}$ mA. Thus, the battery lifetime for the ISN $L_{ISN}$ is

(51) $$\begin{array}{c} L_{ISN}=\frac{E_{battery}}{I_{avg,ISN}} \end{array}$$

in hours. From Figure 12, it can be observed that with certain values of *N*, the battery lifetime of a single ISN is higher than that of a LoRa transmitter-equipped sensor node; it is worth noting that future developments in IRSs can be expected to make this gap between the ISN and a transmitter-equipped sensor node even larger.

**Figure 13:**
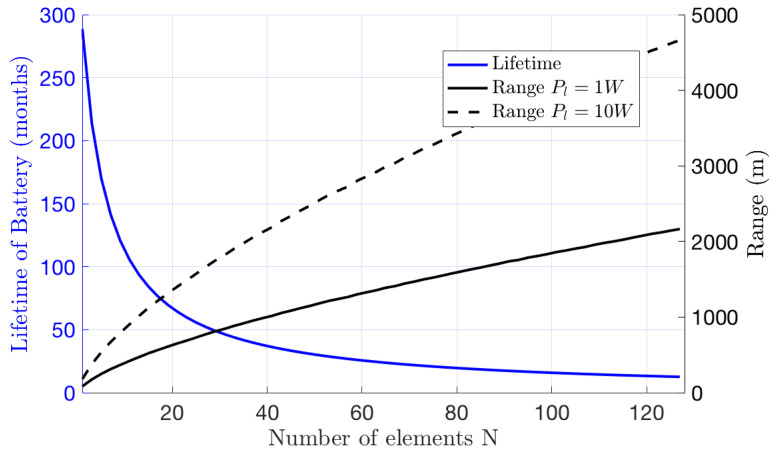
Lifetime and range of a sensor node vs. the number of IRS elements *N*. Note that the range has two different curves, corresponding to different BS transmit powers $P_{l}$. The measurement period is $T_{m}=30$ s.

Next, in Figure 13, we focus on how the battery lifetime of an ISN decreases with increasing *N*, as adding more elements linearly increases the power consumption of the ISN to operate and adjust the IRS phases and deplete the sensor’s battery capacity, as in (50). Moreover, Figure 13 shows the proportional increase in range *d*, which is the distance from the ISN to the user. To construct the relationship between range and number of elements *N*, recall $\beta_{2}^{l}=\frac{10^{-3}}{d^{3}}$ in Table 3; using (13), we can write

(52) $$\begin{array}{cc} \gamma_{l} & =\frac{P_{l}{\left|f_{l}^{H}H_{1,l}\Phi_{l}h_{2,l}\right|}^{2}}{\sigma^{2}} \end{array}$$

(53) $$\begin{array}{cc} & =\beta_{2}^{l}\frac{P_{l}{\left|f_{l}^{H}H_{1,l}\Phi_{l}z_{2,l}\right|}^{2}}{\sigma^{2}}, \end{array}$$

(54) $$\begin{array}{cc} \; & =\left(\frac{10^{-3}}{d^{3}}\right)\frac{P_{l}{\left|f_{l}^{H}H_{1,l}\Phi_{l}z_{2,l}\right|}^{2}}{\sigma^{2}}, \end{array}$$

where $h_{2,l}=\sqrt{\beta_{2}^{l}}z_{2,l}$ and $z_{2,l}∼\mathrm{CN}\left(0,I_{N}\right)$. In terms of *d*, this becomes

(55) $$\begin{array}{c} d={\left(\frac{10^{-3}P_{l}{\left|f_{l}^{H}H_{1,l}\Phi_{l}z_{2,l}\right|}^{2}}{\gamma_{l}\sigma^{2}}\right)}^{1/3}, \end{array}$$

and we can fix $\gamma_{l}=3$ dB; then, $R_{l}\approx 0.8$ (19). It can be seen that increasing *N* expands the sensor’s range. Moreover, increasing the transmitting power of the BS plays a significant role in extending the sensor range, as shown in Figure 13.

**Figure 14:**
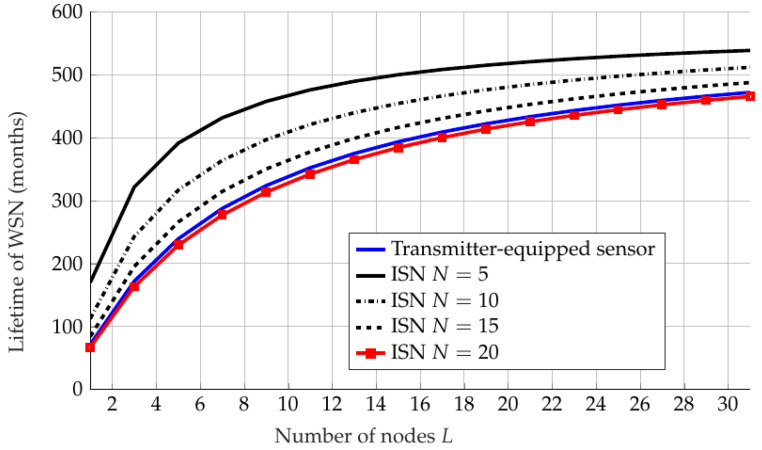
Lifetime of WSN with IRS for different *N* and without an IRS against the number of sensor nodes *L*.

In Figure 14, we simulate and compare the lifetime of a WSN equipped with IRSs vs. a typical WSN without IRSs. We assume fair transmission (such as with RR) for both scenarios and that all sensor nodes are aware of their turn in transmission, and use a measurement period $T_{m}=30$ s. At any period, only one node is active; the other (L−1) nodes are in sleep mode. The total battery energy in the system for *L* sensor nodes is $E_{total}=LE_{battery}$. The average current consumption of the entire network $I_{avg,net}$ can be derived as

(56) $$\begin{array}{c} I_{avg,net}=\frac{1}{T_{m}}\left(I_{tr}T_{tr}+I_{sen}T_{sen}+I_{proc}T_{proc}+I_{s}T_{s}+\left(L-1\right)T_{s}I_{s}\right), \end{array}$$

and the WSN lifetime $L_{WSN}$ in hours can then be found as

(57) $$\begin{array}{c} L_{WSN}=\frac{E_{total}}{I_{avg,net}}. \end{array}$$

Figure 14 shows that for certain values of *N*, i.e., (N=5,10,15), the lifetime of the WSN with IRSs is larger than the WSN without IRSs, which means that the performance is improved under IRSs with constraints on the number of elements in regard to energy consumption. Moreover, with an increasing number of sensor nodes, the lifetime of WSN increases logarithmically due to the discharge current in sleep mode. Note that this simulation assumes that each sensor is scheduled the same number of times, which happens in the scheduling schemes described in this paper for RR, PF, and OS. However, for OS it may or may not happen, given a specific placement in which the receiver is placed equidistant from all sensors.

## 8. Conclusions and Future Directions

In this paper, we formulate an innovative solution for WSN using modulated IRSs. Each sensor is attached to an IRS leading to an IRS-sensor node (ISN), which replaces sensors with active RF equipment. This mitigates the caveat of battery usage in the sensors, as the power for transmission is exported to the BS. First, we define the system model under LoS and Rayleigh channel conditions. We study BPSK transmission from the ISN and provide the system outage probability for different scheduling schemes, including opportunistic scheduling (OS), proportional fairness (PF), and round-robin (RR). Multi-user diversity is investigated and exploited in the OS scheme for different numbers of IRS elements *N* and numbers of sensors *L*. The proposed scheduling schemes are further compared with different metrics, such as outage, minimum throughput, average throughput, achievable rate, and fairness. WSNs with ultra-low power sensors, as in the case of ISNs which do not depend on costly control circuitry, are investigated, and scheduling protocols under this setup are studied as well.

Different scheduling schemes can be best used under different scenarios. For instance, OS is best used if the WSN has a large number of sensor nodes in order to attain multi-sensor diversity gains. On the other hand, in cases involving lack of feedback from the user, the BS can deploy RR scheduling, which is channel independent and fair. Moreover, to find a middle ground between fairness and performance, the PF scheduling scheme can be utilized. The system throughput is best improved with OS as opposed to other scheduling schemes in this system. Future directions include scheduling ISNs without assumed favorable propagation in the system and the subsequent effects on the system performance due to interference between the sensors. An interesting line for future work is to include environmental conditions (such as temperature, dust, weather conditions) in the model and observe their effects the performance in the system. Moreover, the choice of different fading channel distributions, such as Rician fading, can be included in future analysis.

## Figures and Tables

**Table 1:** Important notations for system model.

Symbol	Definition
*M*	Number of BS antennas
*N*	Number of IRS elements
*L*	Number of ISNs
$H_{1,l}\in C^{M\times N}$	LoS BS-IRS *l* channel
$h_{2,l}\in C^{N}$	IRS *L*-user channel
$h_{l}\left(t\right)\in C^{M}$	End-to-end channel
$\beta_{1}^{l}$	Path loss factor for $H_{1,l}$
$\beta_{2}^{l}$	Path loss factor for $h_{2,l}$
$\beta^{l}$	Path loss factor for $h_{l}\left(t\right)$
$a_{l}\in C^{M}$	BS array response vector
$b_{l}\in C^{N}$	IRS *l* array response vector
$\Theta_{l}\left(t\right)$	Phase shift diagonal matrix for IRS *l*
$v_{l}\left(t\right)$	Phase shift vector for IRS *l*
$\theta_{l,n}\left(t\right)$	Phase shift for the $n^{th}$ element of IRS *l*
$G_{l}=H_{1,l}\mathrm{diag}\left(h_{2,l}\right)\in C^{M\times N}$	Reformulation of the channels
$x_{l}$	Reference known signal
$f_{l}\in C^{M}$	Beamforming vector designated for ISN *l*
$P_{l}$	Reference signal power
$\gamma_{l}$	Signal to noise ratio
$X_{l}\left(t\right)$	BPSK signal
$p_{e}$	Cross-coverage probability

**Table 2:** List of abbreviations.

Acronym	Definition
IRS	Intelligent reflecting surface
ISN	IRS sensor node
WSN	Wireless sensor network
BS	Base station
RR	Round-robin
PF	Proportional fairness
OS	Opportunistic scheduling
RF	Radio frequency
IoT	Internet of Things
SWIPT	Simultaneous wireless information and power transfer
LoS	Line of Sight
UPA	Uniform planar array
ULA	Uniform linear array
AoA	Angle of arrival
AoD	Angle of departure
SNR	Signal to noise ratio
CSI	Channel state information
BPSK	Binary phase shift keying
LDPC	Low-density parity check
BER	Bit error rate

**Table 3:** Simulation parameters.

Parameter	Value
**Array** **Parameters**	
BS configuration	Uniform linear array
IRS configuration	Uniform planar array
Antenna gain	5 dBi
$d_{BS}$, $d_{IRS}$	$0.5\lambda_{c}$
Noise level	−30 dBm
**Path Loss**	
Model	$\frac{10^{-C/10}}{d^{\alpha }}$
*C* (Fixed loss at d=1 m)	25 dB ($\beta_{1}^{l}$), 30 dB ($\beta_{2}^{l}$)
α (Path loss exponent)	2.2 ($\beta_{1}^{l}$), 3 ($\beta_{2}^{l}$)
**System Dimensions**	
(L,N,M)	(32,32,64)
Time slots *t*	2000

**Table 4:** Simulation Parameters.

State	Time Duration (s)	Power Consumption (mW)
Sensing unit	$T_{sen}=0.025$	10.5
Transmitter	$T_{tr}=0.0065$	92.4
Processor	$T_{proc}=0.0335$	1.8

**Table 5:** Battery characteristics.

Characteristics	Value
Sensing unit voltage	$V_{s}=2$ V
Transmitter and processor voltage	$V_{b}=3.3$ V
Battery capacity	C=950 mAh
Self-discharge current	$I_{s}=7.5\times 10^{-3}$ mA

## Data Availability

Not applicable.
